# Sensitivity of Neural Responses in the Inferior Colliculus to Statistical Features of Sound Textures

**DOI:** 10.1016/j.heares.2021.108357

**Published:** 2021-10-14

**Authors:** Ambika P. Mishra, Fei Peng, Kongyan Li, Nicol S. Harper, Jan W. H. Schnupp

**Affiliations:** a Department of Neuroscience, City University of Hong Kong, Hong Kong SAR; b Department of Physiology, Anatomy and Genetics, University of Oxford, Oxford, UK

**Keywords:** Time-averaged statistics, sound texture, inferior colliculus

## Abstract

Previous psychophysical studies have identified a hierarchy of time-averaged statistics which determine the identity of natural sound textures. However, it is unclear whether the neurons in the inferior colliculus (IC) are sensitive to each of these statistical features in the natural sound textures. We used 13 representative sound textures spanning the space of 3 statistics extracted from over 200 natural textures. The synthetic textures were generated by incorporating the statistical features in a step-by-step manner, in which a particular statistical feature was changed while the other statistical features remain unchanged. The extracellular activity in response to the synthetic texture stimuli was recorded in the IC of anesthetized rats. Analysis of the transient and sustained multiunit activity after each transition of statistical feature showed that the IC units were sensitive to the changes of all types of statistics, although to a varying extent. For example, we found that more neurons were sensitive to the changes in variance than that in the modulation correlations. Our results suggest that the sensitivity of the statistical features in the subcortical levels contributes to the identification and discrimination of natural sound textures.

## 1 Introduction

Sound textures are the collective result of many related acoustic events, and an influential psychoacoustic study has indicated that many natural sound textures are largely characterized by key statistical features (McDermott & Simoncelli, 2011). Thus, textures with one particular set of statistical features will sound like a crackling fire, and textures with another set may sound like a rushing stream or a swarm of insects. McDermott & Simoncelli (2011) developed a model which extracts such key statistical parameters from recordings of natural sound textures, and they hypothesized that these types of statistics are likely also measured by the brain along successive stages of neural processing in the auditory pathway, and may be used in the identification and discrimination of different types of natural sounds.

The McDermott & Simoncelli (2011) model comprises two bandpass filter stages. The first set of filters was designed to approximate cochlear processing, and the envelopes were computed from each filter. The marginal moments and cochlear correlations are computed from the envelopes of the first-stage filters. The signal envelopes computed in the first filter set are then also passed through a second, modulation filter bank, and modulation power and modulation correlation statistics are calculated over the output of these second-stage filters. The bandwidths and frequency ranges used in the filter banks are chosen in agreement with known frequency and modulation tuning properties seen at the level of cochlea and midbrain neurons respectively. The set of statistical parameters computed by the model are thought to uniquely identify textures, and they place each natural sound texture in a very high dimensional parameter space. The value of these parameters has been demonstrated empirically by McDermott & Simoncelli (2011), who were able to show that artificial textures synthesized to match a given statistical parameter set can in most cases be easily identified as a particular texture type, and sound remarkably “life-like”. However, the number of parameters generated by McDermott & Simoncelli’s original to characterize a given texture is very large, perhaps up to several thousand depending on the number of frequency and modulation channels used, and this large parameter set is highly redundant. In a previous study (Mishra, Harper, & Schnupp, 2020), we reported that a large portion of the variance in these very high-dimensional texture spaces can be captured by a much lower number of components, which often lend themselves to intuitive interpretation. Thus, marginal moments mostly distinguish sound textures along dimensions of “sparseness” or “burstiness”, which discriminate textures according to the extent to which they exhibit intermittent bursts of sound energy, instead of a more smooth, continuous sound delivery. Meanwhile, cochlear correlation distinguishes “highly correlated” textures, such as applause, from “poorly correlated” ones, such as the sound of bubbling water. Modulation power mostly differentiates “rapidly modulated sounds” (e.g. buzzing sounds of the wings of bees) from “slowly modulated sounds” like ocean waves. Finally, modulation correlations on the other hand can differentiate sound textures that have sudden “phase-changes” or onset-offset-like mechanisms (e.g. bomb explosion, firecrackers). While operating in this reduced statistical parameter space ignores some of the richness and subtlety of natural sound textures, it does make it easier, for example, to select relatively small stimulus sets for experiments in a manner that can be deemed fairly representative of the variety of textures likely found in the environment.

These different types of statistics, envelope marginals, cochlear correlations and modulation parameters, can also be thought of as forming a “hierarchy”, given that sound envelopes in each cochlear frequency band must be extracted first before cochlear correlations and amplitude modulations can be computed. Similarly, one might expect this hierarchy to be emerge gradually along the ascending auditory pathway. The auditory brainstem might be able to measure marginals by observing the activity of groups of auditory nerve fibers within just a narrow range of characteristic frequency band individually, but the computation of cochlear correlations requires information to be combined across many frequency channels along the tonotopic array. Measuring modulation statistics requires a second filtering step which is needed for neither marginals nor cochlear correlations, so perhaps sensitivity to these statistics only emerges relatively late, perhaps at the level of the midbrain or even later. This notion of a “hierarchy”, and the types of statistical features chosen by McDermott & Simoncelli (2011) were motivated at least in part by known physiological properties of neurons in the auditory pathway, including modulation tuning (Hsu, Woolley, & Fremouw, 2004; Joris, Schreiner, & Rees, 2004; Miller, Escabí, Read, & Schreiner, 2002; Rodríguez, Chen, Read, & Escabí, 2010), and the sensitivity to temporal coherence (Elhilali, Ma, Micheyl, Oxenham, & Shamma, 2009; Krishnan, Elhilali, & Shamma, 2014). Furthermore, McDermott & Simoncelli (2011) hypothesized that the sensitivity to each of these types of statistical features may already be present at the level of the auditory midbrain, but the extent to which neurons in the inferior colliculus (IC) are already sensitive to each of these types of statistical features has not yet been examined experimentally.

The objective of this study is to explore how pervasive sensitivity to each of these statistical feature types is at the level of the IC. If IC neurons are sensitive to a particular statistical sound texture feature, then changes in neural responses should be observed whenever that particular feature of a sound texture changes abruptly, but all other characteristics are held constant. In contrast, if a neuron is deaf to that particular type of statistical feature, then its response should remain unchanged. To determine how common sensitivity to each of the types of statistical features is among IC neurons, we therefore recorded extracellular responses of IC multiunits with silicon array electrodes implanted into the IC of ketamine/xylazine anesthetized female Wistar rats to sets of texture stimuli, which were synthesized to incorporate, at specific time points, abrupt changes in just one type of statistical feature while leaving all other stimulus parameters unchanged. The recordings were examined for either transient or sustained changes in neural activity evoked by changes in each type of statistics. The results show that sensitivity to all types of texture statistics can already be observed at the level of the IC, although to a varying extent.

## 2 Materials and Methods

### 2.1 Animal subjects

Five young adult (eight weeks old) female Wistar rats weighing approximately 250 − 280g were used for the terminal IC recording experiments described here. All rats were purchased from the Chinese University of Hong Kong. The experimental procedures in the study were approved by the Ethics Sub-Committee on the Use and Care of Animals at the City University of Hong Kong and under license by the Department of Health of Hong Kong [Ref. No. (18-167) in DH/HA and P/8/2/5 Pt.5].

### 2.2 Stimulus design

#### 2.2.1 Selection of representative sound textures

**Figure 1:**
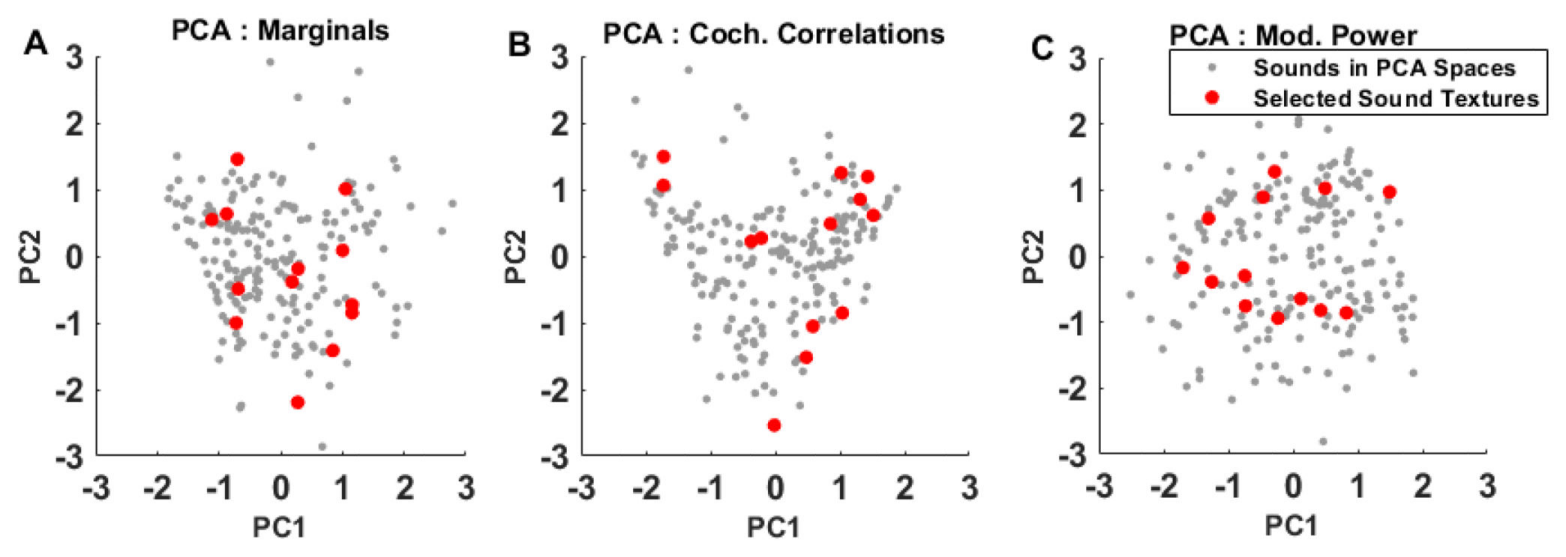
Selecting representative sound textures from a corpus of natural sound recordings. Sound textures in the PCA space of (A) Marginal statistics, (B) Cochlear correlation statistics, and (C) Modulation power statistics. x- and y-axis represents the first and second principal components respectively. Grey dots represent sounds in the corpus. Red dots represent the selected texture sounds.

A set of 13 sound texture recordings was chosen from our corpus of 200 recordings. The selection was essentially random, but we visualized the coordinates of the selected sound textures in the “principal component (PC) space” of statistical texture parameters described in our previous study (Mishra et al., 2020) to make sure that the random selection is reasonably “representative”. For the entire corpus, marginals, cochlear correlations, and modulation power statistics of each sound texture had been measured separately and subjected to principal component analysis (PCA) to allow us to visualize the location of each chosen sound along principal component coordinates within the corpus. Figure 1 shows the coordinates of the chosen sounds along the first two principal dimensions for each feature type in PC space. By inspecting the coordinates of the chose sounds relative to the rest of the corpus we can verify that the textures selected for the current study are widely distributed, and cover a substantial part of the parameter space spanned by the corpus without leaving large parts of texture space unsampled. In this sense, the 13 selected textures can be considered “representative samples”. The selected 13 textures included the following sounds: “applause”, “barn swallow calls”, “cackling geese”, “church bells”, “burning wood sticks”, “fireworks”, “foot-steps in water”, “frogs at night”, “galloping horses”, “stirring liquid in a glass”, “lawnmower”, “xylophone”, and “tin can”.

#### 2.2.2 Synthesized stimuli

**Figure 2:**
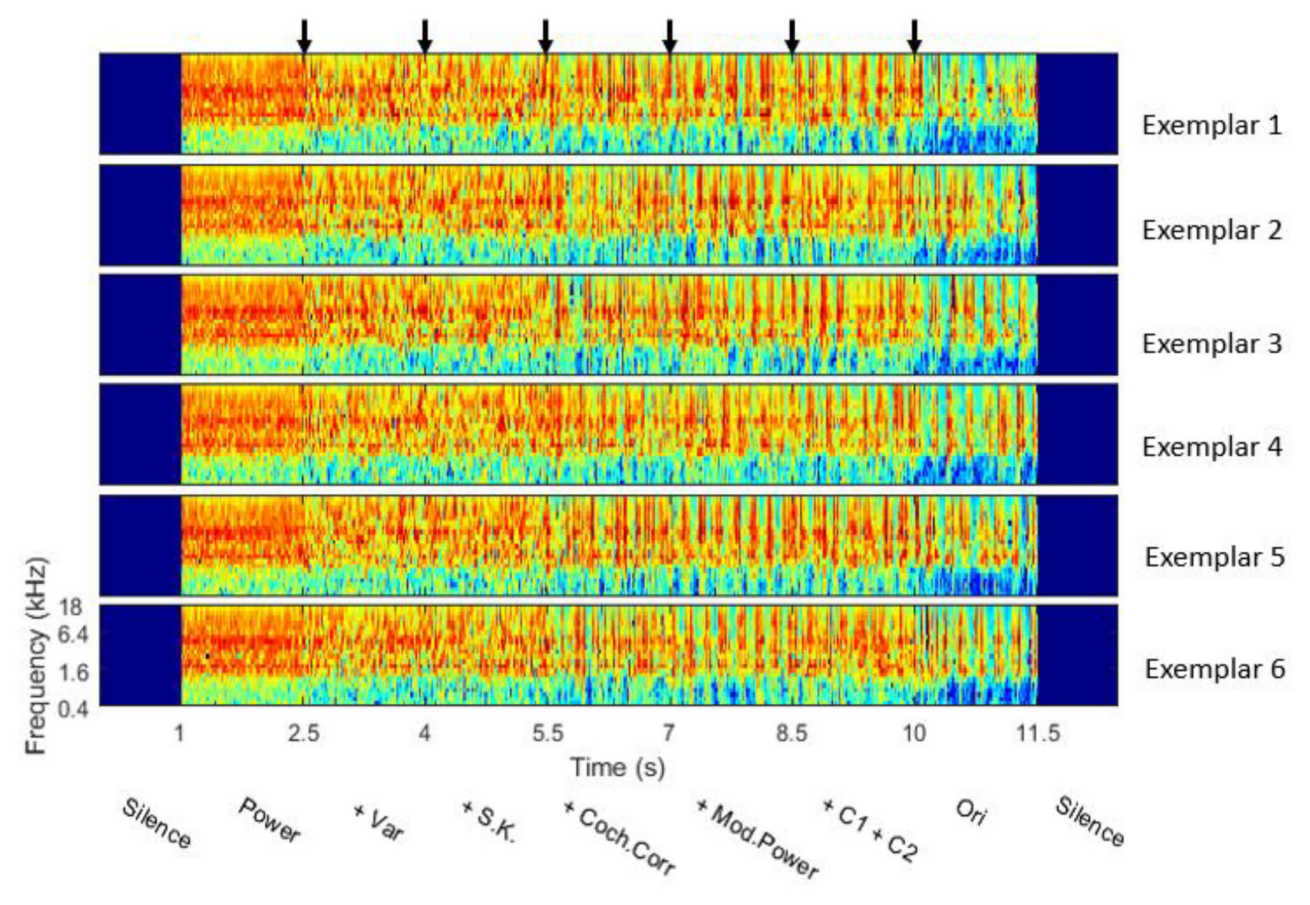
Stepwise morphing of spectrally shaped noise towards full sound textures, starting from different random seed values. Cochleagrams for 6 exemplars of synthesized sound (stirring liquid in a glass) using 6 random seeds of Gaussian white noise. The arrows in the top of the figure represent the transition time of the synthetic variants. From left to right, the synthetic variants correspond to Power, +Var, +S.K., +Coch.Corr, +Mod.Power, and +C1+C2, and then end with the original sound Ori.

**Figure 3:**
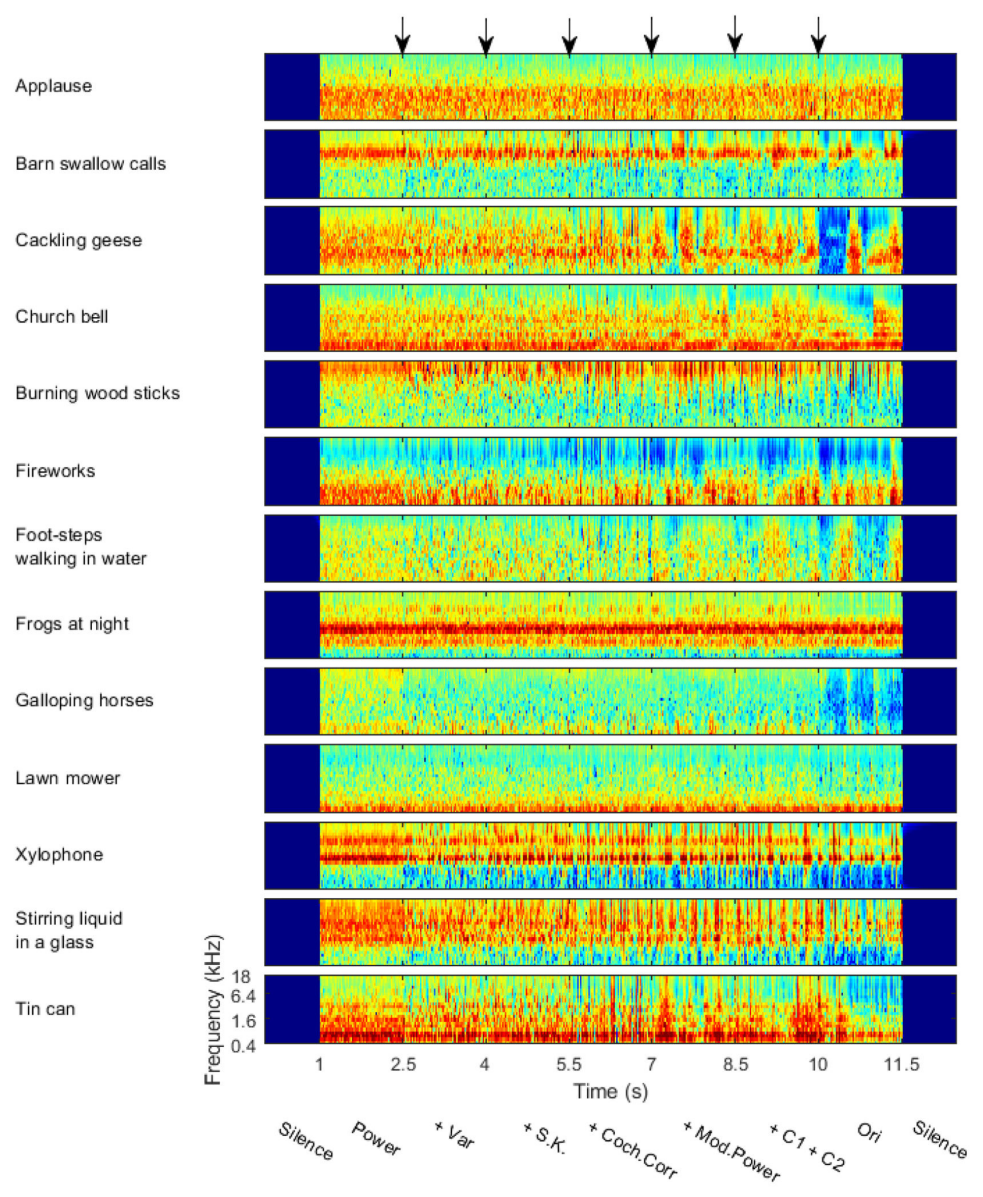
The stimulus set used to characterize statistical feature sensitivity in the IC. Each row shows the cochleagrams for one exemplar of our stimulus set, morphing towards the sound texture indicated at the left. The arrows mark the transition times. From left to right, the synthetic variants correspond to Power, +Var, +S.K., +Coch.Corr, +Mod.Power, +C1+C2, and Ori.

For each of the 13 chosen textures, we used the Sound Synthesis ToolBox V1.7 (McDermott & Simoncelli, 2011) to synthesize sound samples which morphed white noise into full-fledged textures in a stepwise process. Each step representing a sudden transition where just one set of statistical parameters changes from that of white noise to that of the appropriate texture. We generated six synthetic variants by matching a subset of the statistics of white noise to the statistics of the original texture. Starting with pseudorandom Gaussian white noise generated with a particular random seed value, the following statistical features were sequentially incorporated in the synthetic variants: the sound power spectrum (Power), variance (+Var), skew and kurtosis (+S.K.), cochlear correlation (+Coch.Corr), modulation power (+Mod.Power), and modulation correlation (+C1+C2). Segments of 1.5 s duration of each of these consecutive synthetic variants were then concatenated with a 10 ms cosine ramp cross fade. In addition, a 1.5 s long segment of the original sound (Ori), selected at random from the recording, was appended at the end, to produce an 11.5 s long stepwise morph which transitions from spectrally shaped noise to the full, natural sound in 7 transitions. To minimize possible idiosyncratic influences that the choice of random seed might have, we produced 6 such morphs with different random seed values for each of the 13 chosen textures. Figure 2 shows the cochleagram of the 6 exemplars generated in this manner for one such sound texture (stirring liquid in a glass). Figure 3 shows the cochleagram of 13 textures. All morphs were scaled to have the same RMS power throughout. The 1.5 s duration of each segment should be long enough to allow neural responses to achieve a new steady state before the next transition, thereby allowing us to quantify both “onset” and “sustained” responses of neurons to each segment, while being short enough that responses to large numbers of segments and transitions can be recorded in feasible experimental durations.

These sound stimuli were presented to the anesthetized rats via AS02204MR-N50-R (PUI audio, Dayton, USA) earphones, coupled to hollow ear bars that were inserted into each ear canal, and driven by TDT (Tucker-Davis Technologies System III) digital signal processor hardware. The acoustic system was calibrated using a microphone (GRAS46DP), and an FIR filter compensated the acoustic signal of the speaker to deliver a flat response across frequencies (0.5 to 20 kHz). The sound level of each morph was normalized to 80 dB SPL prior to splicing the stimuli together. The sampling rate of the stimuli was 48,828.125Hz. Each of the 13 textures times 6 exemplars was presented 10 times, for a total of 13×6×10 = 780 stimuli, the textures were presented in random order to characterize neural responses.

### 2.3 Electrophysiological recordings

To check the hearing status of our experimental animals prior to the experiment, we tested Preyer’s reflexes and performed a physical examination of the ears and the tympanic membrane. The rats were anesthetized by i.p. injection with an initial induction dose of a mixture of ketamine (80 mg/kg) and xylazine (12 mg/kg). For maintenance of anesthesia during electrophysiological recordings, a syringe pump delivered an i.p. infusion of 0.9% saline solution of ketamine (17.8 mg/kg/h) and xylazine (2.7 mg/kg/h) at a rate of 2.1 ml/h. Body temperature was measured rectally and maintained with a heating pad (RWD Life Science, Shenzhen, China) and blanket at 38°C both during surgery and recording. The state of the animal was monitored (temperature, and toe-pinch withdrawal reflexes) throughout the experiment. The animal was placed inside a sound-attenuating chamber, and head fixed using hollow ear bars in a stereotactic frame (RWD Life Sciences).

Auditory brainstem responses (ABRs) were recorded to evaluate the hearing sensitivity of animals before surgery. ABRs were evoked by the clicks (500μs white noise pulses) at a rate of 23Hz, and 400 click presentations were played at each intensity level (30 dB SPL to 80 dB SPL in 5 dB steps). The clicks were played through the hollow ear bars using custom-made headphone drivers based on AS02204MR-N50-R (PUI audio, Dayton, USA). Stainless steel needle electrodes placed at the mastoids, nose, and back. The ABR corresponded to the averaging scalp potentials between mastoid and the vertex of the rat’s head (Rosskothen-Kuhl, Buck, Li, & Schnupp, 2021). ABR thresholds at 30 dB SPL or below were deemed normal.

For the IC recording, a craniotomy was performed to the right of the midline just anterior to lambda, and single shank 32-channel (50 μm spacing between recording sites, ATLAS Neuroengineering, E32-50-S1-L6) silicon electrodes were inserted into the IC in a dorsal-ventral direction through the overlying cortex. We confirmed the location of the electrodes in the IC based on physiological criteria, such as strong click and tone evoked responses at very short response latencies of 7 ± 2 ms (mean ± standard deviation). Based on these short response latencies, we suspect that most of our recording sites were in the central region of IC, given that neural responses from the dorsal cortex and external cortex of IC typically exhibit longer latencies of 15 ms or above (Syka, Popelář, Kvašňák, & Astl, 2000). The neural signals recorded from the electrodes were amplified by a PZ5 preamplifier and recorded at a sampling rate of 24,414 kHz with a RZ2 system (Tucker-Davis Technologies).

### 2.4 Data analysis

The aim of this paper is to assess the extent to which neurons in the IC are sensitive to the types of statistical features that are thought to be important in distinguishing auditory textures. We hypothesized that such a sensitivity should manifest in changes in neural activity following changes in statistical features of the morphed stimuli. Such changes in neural activity could be either transient or sustained. To look for transient changes we analyzed a short, 50 ms wide time window immediately after each stimulus transition, while for the sustained response analysis we considered time windows from 0.5 to 1.5 s after the transition. These windows were chosen by inspection of a large number of PSTHs which indicated that IC neurons often exhibited brief transient responses, and that 0.5 s is typically plenty of time for IC neurons to settle into a “steady state” after changes in stimulation.

Neural activity was analyzed offline using an “analog measure of multiunit activity” (aMUA) measure, which measures the voltage signal power in the frequency band corresponding to the extracellularly recorded action potentials. The raw signal recorded from the electrodes is first bandpass filtered between 300 and 6000 Hz by a zero-phase shifting Butterworth filter. We then took the absolute value of the filtered signal, and downsampled it to 2 kHz. This method for quantifying neural activity is identical to that used in previous studies (Kayser, Petkov, & Logothetis, 2007; Sadeghi, Zhai, Stevenson, & Escabí, 2019; Schnupp, Garcia-Lazaro, & Lesica, 2015).

#### 2.4.1 Analyzing transient neuronal responses

**Figure 4:**
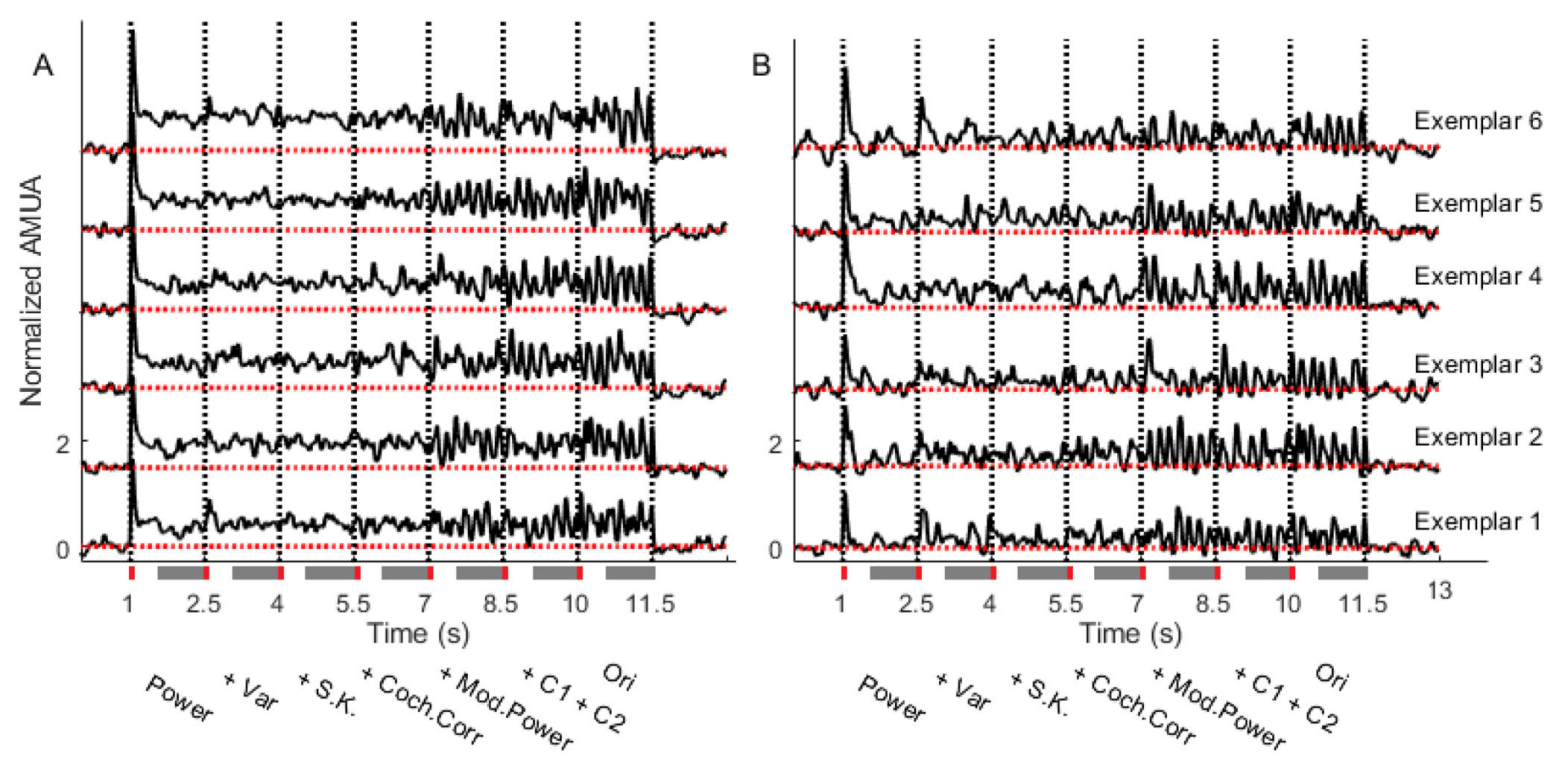
Examples of two multiunits in response to 6 exemplars of the texture (Cackling Geese). Each row represents the response to one exemplar of the stimulus, the black dashed lines represent the transition time of the synthetic variants, and the red dashed lines represent the AMUA baseline. Red and gray bars indicate the time window of the transient response and sustained response, respectively.

One potential difficulty in the analysis of these data is that the response properties of the IC neurons recorded from are not fully known, and that they are likely to exhibit transient responses to some idiosyncratic spectro-temporal features of a given sound texture. If such features happen to occur by chance near one of the statistical parameter transitions, then such responses to spectro-temporal features could be misinterpreted as responses to the statistical parameter transitions. Below we will see examples (consider Fig 4 below) of such strong fluctuations in responses which are stimulus driven but most likely unrelated to statistical parameter transitions as they happen sporadically throughout the steady-states of the texture morphs. A good statistical test for the significance of responses that are time-locked to the statistical parameter transitions must therefore be able to quantify whether response amplitude changes around the transition are larger than would be expected given the potentially large “background” fluctuations in responses caused by other stimulus related features. Conventional parametric tests are unsuitable for this, not just because the distributions of response amplitudes will often deviate from normal, but also because response fluctuations occur in a “nested” fashion: a response to a sound feature that just happened to occur close to a transition in one particular exemplar would be expected to occur in most of the 10 repeats of that particular exemplar, but that similar transition unrelated responses happen at the same point in time in other exemplars or texture types is probably less likely. The trial type therefore matters and one cannot consider the responses around each transition as a separate independent sample. To assess the statistical significance of response amplitude changes around stimulus transitions we therefore constructed resampling tests which are designed to compare response changes observed at transitions against “null distributions” of changes seen at non-transition points in data which were resampled in a manner that preserved the nested structure.

In practice, this test assessed whether the absolute difference between the mean response amplitudes during the 50 ms just before and just after the stimulus transition was larger than would be expected given the computed null distributions. Furthermore, to be able to interpret significant, transition-evoked changes in neural responses as evidence that the neurons are sensitive to the given type of statistical feature, we also require that such a significant responses “generalize”, that is, that they must occur for several of the different texture types tested here. Significant responses to a given statistical parameter change for only a single one of the thirteen texture types tested would provide unconvincing evidence for a general sensitivity to that statistical feature, even if that one transition response was individually highly statistically significant. Ideally we would like to see significant responses for many, if not all texture types, but given that the texture types are by design very different, we need to allow for the fact that some textures may not drive a particular set of neurons very reliably, which would make them less suitable for revealing significant changes at transition points even if the neurons are in principle sensitive to the feature in question. We therefore carried out our resampling test separately for each of the 13 textures, and then applied the criterion that, for a given type of feature transition, a multiunit would have to exhibit significant responses to at least four of the 13 textures tested to be considered sensitive to this statistical feature. We also conducted a control analysis to verify that this test and the criterion ensure a high degree of specificity.

To judge whether the change in mean neural response amplitude during 50 ms on either side of a stimulus transition is larger than expected by chance, we averaged responses over each of the 10 repeats of each exemplar and over each of the 6 exemplars of each texture, and computed the absolute differences in these mean responses to compute the “true transition response”. We then we used a bootstrap method to estimate the expected null distribution for this response measure. We resampled the neural response time series during a steady state response period from 1000 ms to 100 ms prior to the transition. Neural response time series during this steady state response (sampled in 10 ms bins) were averaged over stimulus repeats to yield a 6 exemplar by 90 time bin neural response matrix. To generate one “simulated null transition response” we picked, uniformly and independently, one random “simulated transition” time point for the mean response to each exemplar, computed the average responses during the 50 ms before and after that time point, and calculated their absolute difference. These bootstrap samples of absolute differences were averaged over the 6 exemplars to generate one simulated null transition response value. This process was repeated 1000 times to generate a distribution of simulated null transition responses, and the p-value of the true transition response was computed as the percentile of the true transition response value in the distribution of null transition values. To be deemed to exhibit a significant transition response, a multiunit had to yield p-values 0.05 for at least 4 of the 13 textures.

To verify that this procedure is highly selective and generates very few false positives, we conducted the following control: We simply replaced the true transition response value (which compares 50 ms before the transition against 50 ms after a transition) with a “false” transition value which compares the response 50 ms before the transition against the response observed during the period from 100 to 50 ms prior to the transition. These “false transition responses” were then compared against the bootstrapped simulated null transition response distribution to compute “sanity check p-values”, which would have to be attributable to false alarms. These sanity-check p-values were subjected to the same criterion of requiring at least four values below 0.05 to fulfil our significance criterion. We conducted this test on all 6 stimulus transitions and all 480 multiunits in our sample, and we obtained only one single false positive result on a single transition for a single multiunit. This low false positive rate (1 false positive in 2880 tests) demonstrates the high specificity of our test.

#### 2.4.2 Measuring sustained neuronal responses

The method that we described for testing the statistical significance of any observed neural response for transient responses cannot be applied directly for sustained response analysis as there aren’t sufficiently many wide time windows to allow adequate resampling. Therefore, we developed a slightly different analysis method for the sustained response analysis.

For estimating the response before a given stimulus transition, we averaged the AMUA amplitudes in a 1 s wide time window just prior to the transition. For each texture, we resampled the averaged AMUA over the 6 exemplars and 10 trials with replacement, and then calculated the mean of these 60 numbers. Repeating this process 1000 times, we obtained a bootstrap distribution of the mean AMUA amplitudes during one second preceding the transition. We then repeated this procedure for a “sustained response period” from 0.5 s to 1.5 s post transition. Finally, we considered the pre- and post-transition bootstrap distributions significantly different at an alpha of 0.05 if they did not overlap by more than 5%. As for the transient responses, we required that a multiunit shows significant differences in the pre- and post-transition sustained responses for at least 4 out of our 13 textures for that multiunit to be considered sensitive to the statistical features that changed during that particular transition.

### 2.5 Spectro-temporal receptive field (STRF) model analysis

To investigate whether observed responses to statistical parameter transitions could be explained by simple spectro-temporal tuning, we built a linear-nonlinear (LN) model (Lohse, Bajo, King, & Willmore, 2020; Rabinowitz, Willmore, Schnupp, & King, 2011). The model approximates the relationship between a ‘cochleagram’ of the sound stimuli and each multiunit’s response, using a linear receptive field, known as a spectrotemporal receptive field (STRF), followed by a simple sigmoidal output non-linearity. A cochleagram is an approximation the representation of sound by the auditory periphery; we used the spec-log cochleagram model from Rahman et al. (2020) but with 20 ms Hanning windows overlapping by 10 ms, and 24 frequency channels. The STRFs used 100 time bins (1000 ms) of the immediate past cochleagram activity to estimate the AMUA amplitude at a given time bin.

For each unit, an LN model was fitted to the AMUA amplitude averaged in 10 ms bins, via minimum mean squared error (MSE), using k-fold cross-validation. To prevent overfitting, the parameters were regularized using L1-norm (LASSO) regularization, the strength of which was governed by a parameter lambda. We divided the neural responses of the unit into three non-overlapping sets; a training set consisting the responses to four exemplars of all 13 stimuli, and a validation set and test set each consisting the responses to one exemplar of all 13 stimuli. We then fit the LN model to the training set for a range of lambda values, and then found the lambda that gave the lowest MSE on the validation set. Then, we again fit the LN model using this lambda to the combined validation and training set. Finally, we used this model fit to predict the response for the test set and we evaluated this prediction using the Pearson correlation coefficient between the prediction and the actual neural response. This procedure was repeated six times, each time with a different validation and test set, enabling the response to all the stimuli to be predicted. The final goodness of the LN predictions were evaluated by the average of the correlation coefficient over the six folds. This correlation coefficient, also averaged over all over units, was 0.54.

### 2.6 AMUA change index

The AMUA change index provides a measure of how much the neural responses change when there is a transition from one synthetic variant to another. To do this, for each unit, we first average the neural responses over all 13 textures and 6 exemplars to provide a single trace over time across the synthetic variants. We then measured the AMUA change index over the two successive synthetic variants by the formula below:

$$\;AMUAChangeIndex\;=\frac{AMUA_{post\;}-AMUA_{pre\;}}{AMUA_{post\;}+AMUA_{pre\;}},$$

where *AMUA*_*pre*_ and *AMUA*_*post*_ represents the averaged AMUA in the pre- and post-transition time window, respectively. The pre-transition time window is 1 s before the transition, and post-transition is 0 to 50 ms (transient response) or 500 ms to 1500 ms (sustained response) post the transition.

We calculated the AMUA change index for both the actual neural data and for the LN predictions. Then for each transition, we compared the distribution of the AMUA change index over the population of units for the actual neural data with the distribution for the LN predictions. We did this for both the transient response and the sustained response.

### 2.7 Computing mutual information (MI) of sustained neuronal responses

The main question of this paper was to identify the extent to which IC neurons are sensitive to changes in the statistical features that distinguish different sound textures. A somewhat related question is whether neural responses themselves may distinguish different texture types, independently of which particular exemplar of a type is being presented. While our stimulus set has not been optimized to address this question, it is perhaps nevertheless of some interest to compute the MI between texture type and neural responses in our dataset. We therefore computed the MI between AMUA amplitude and sound texture independently for each multiunit, and asked how the MI values change as more and more texture features were added in the synthetic variant. We averaged the AMUA amplitudes over a 1 s wide steady-state response window just before the next transition for each trial, yielding 60 mean AMUA response amplitudes (6 exemplars x 10 trials per exemplar). We discretized these AMUA amplitudes into six levels, and used the adaptive-direct method (Buck, Rosskothen-Kuhl, & Schnupp, 2021; Itskov, Vinnik, Honey, Schnupp, & Diamond, 2012; Nelken, Chechik, Mrsic-Flogel, King, & Schnupp, 2005) to estimate MI between single trial AMUA amplitude and the identity of the texture type (1-13 possible textures). We bias-corrected MI-values and determined whether MI values were significantly greater than zero (α = 0.01) using the permutation method described in Nelken et al. (2005). This involved subtracting bias estimates obtained by reshuffling the response and stimulus labels 100 times. MI values were calculated separately for each synthetic variant (i.e. each synthesis step of our morphs), and the distributions of MI values were examined for units (n = 387) included if they showed the corrected MI values significantly larger than zero for all synthetic variants.

## 3 Results

In total 480 multiunits were recorded in the course of 15 penetrations with the 32 channel multielectrode (3 penetrations / animal). Figure 4 shows examples of average AMUA responses from two representative multiunits to the 6 exemplars of one particular texture morph. Both multiunits show an increase in response at the transition from the Power to the +Var condition, but for the unit in Figure 4A this response appears sustained, while for the unit in Figure 4B it seems entirely transient. Both multiunits also show much more variable responses after the transition from the +Coch.Corr to the +Mod.Power condition. Also, as expected, we see that the response patterns across the 6 exemplars, which have identical statistical texture parameters but different random seeds, are similar but not identical.

**Figure 5:**
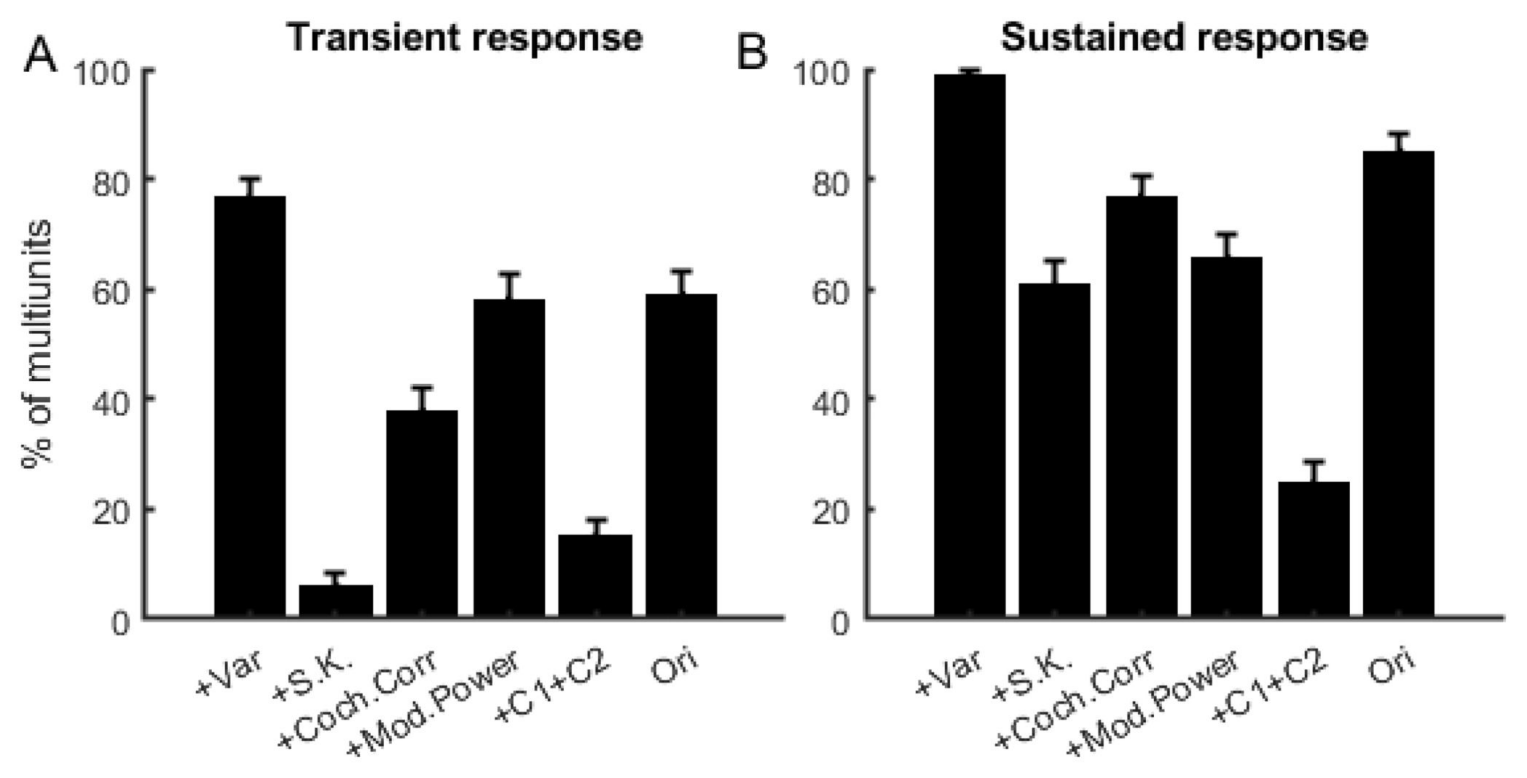
The percentage of multiunits in the IC showing significant changes in transient response (A) and sustained response (B) across synthetic variants. Error bars represent 95% Wilson confidence intervals.

We used the bootstrap methods described in section 2.4 to identify which transitions in texture statistics evoked either transient or sustained changes in the neural responses across our dataset of 480 multiunits. The proportion of multiunits which were sensitive to each of the texture feature types as shown graphically in Figure 5A for transient and in Figure 5B for sustained responses. We found that 76.46% of multiunits gave significant transient responses at the +Var transition, 6.04% at +S.K, 37.71% at +Coch.Corr, 58.13% at +Mod.Power, 15% at +C1+C2, and 58.96% at +Ori, respectively. For the sustained response, the proportions were 98.96% at +Var, 60.62% at +S.K., 76.88% at +Coch.Corr, 65.62% at +Mod.Power, 24.58% at +C1+C2, and 85% at +Ori, respectively. Sensitivity to all the statistical features proposed by McDermott & Simoncelli (2011) was therefore widespread among IC neurons. Note also the large proportions of multiunits which showed significant changes in their responses in the final +Ori transition, which indicates that many multiunits are sensitive to other features of the recorded sounds which are not captured by in synthetic textures created with the McDermott & Simoncelli (2011) Sound Synthesis Toolbox.

**Figure 6:**
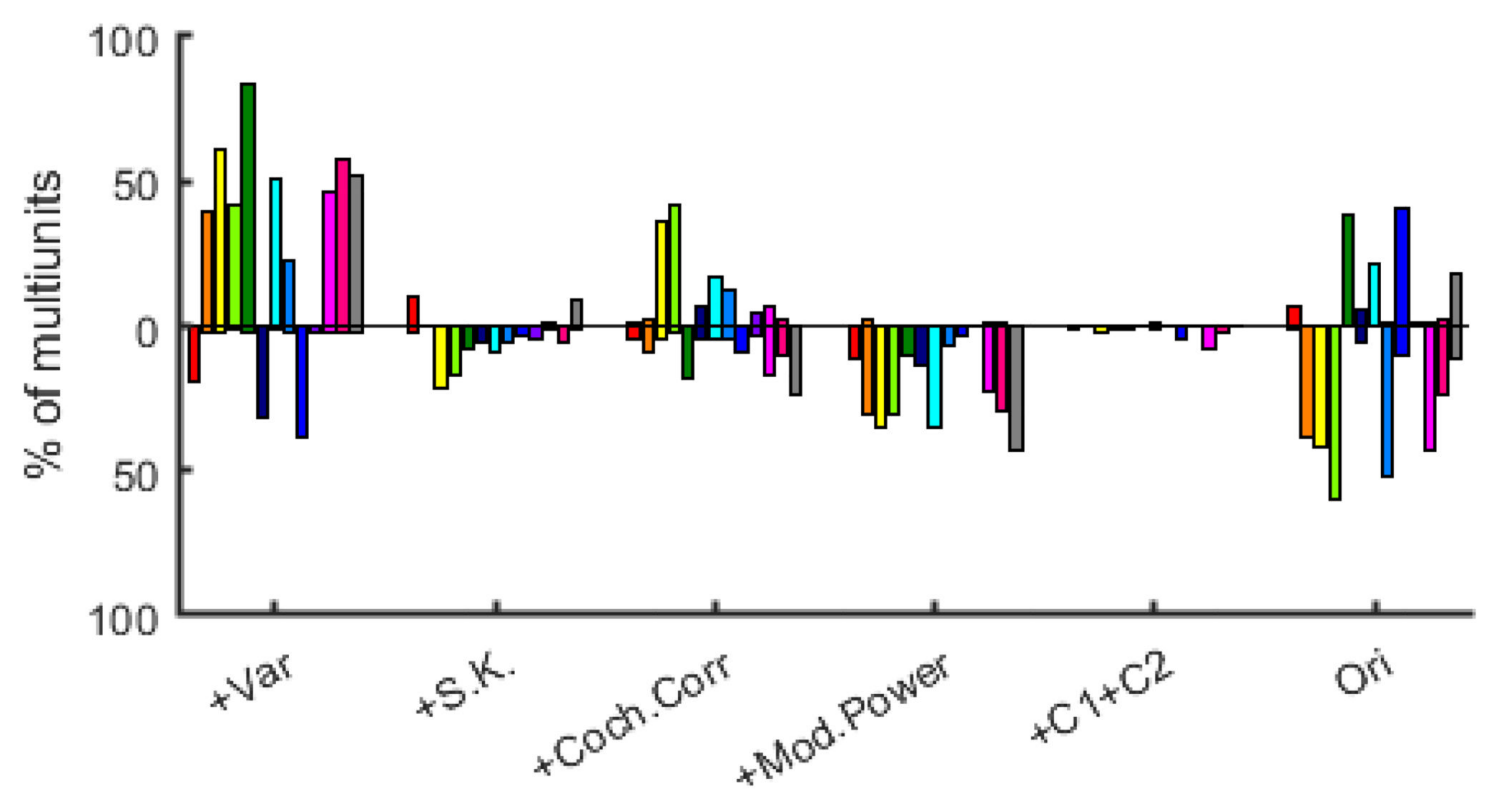
The percentage of multiunits showing significant increases or decreases respectively in their sustained responses after each of the feature transitions shown on the x-axis. Different colors bars show the results for each of the 13 different textures, and the textures are in the same order in Figure 3.

Sustained changes in sustained responses could manifest as either increases or decreases in firing, and these are not distinguished in Figure 5B. To examine whether particular changes in statistical features were more likely to result in increasing or decreasing firing rates we plot in Figure 6, separately for each of our 13 textures, the proportion of IC multiunits where a given transition resulted in significant increases and/or decreases in response strength. One observes curious trends. For example while the +Var transition in most cases led to increases in mean firing rates, for +S.K. and +Mod.Power, decreases were more common, and for the other transitions, the picture was mixed.

**Figure 7:**
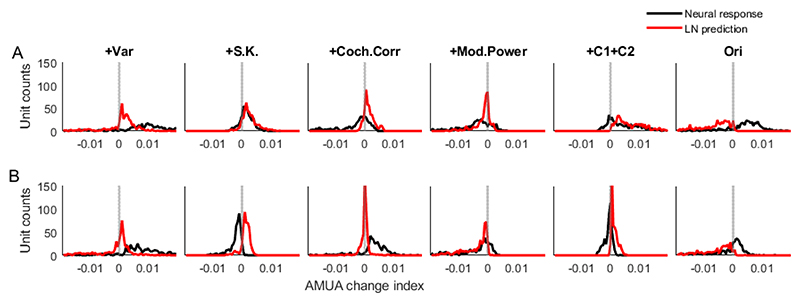
The distribution of the AMUA change index of transient (A) and sustained (B) response for each of the feature transitions.

To examine the relationship between the change of neural response and the change of log spectrogram of the stimulus we used a linear-nonlinear (LN) model fitted to the responses of each neuron. We used the fitted LN model predict what the neural responses would be if they just depended in a linear manner (with a simple static nonlinearity) on the sound spectrogram – that is, we asked how much of the sensitivity to statistical change that we see can be explained by the spectrotemporal tuning of the neurons. To this end, we compared a distribution of a measure of response change (AMUA change index) over units from the actual recording and LN predictions for each transition (Figure 7). For most recorded units, both the actual recordings and the LN predictions showed an increased response at the +Var transition. However, the AMUA change index for most units was smaller for the LN predictions was smaller than that for the actual recording (Figure 7A, first column). We also observed that the AMUA change index for the actual recording is opposite from that for the LN predictions for some transitions. For example, at the +S.K. transition, most units from the actual recording showed a decreased response (Figure 7B, second column), however, most units from LN predictions showed an increased response. At the transition to Ori, most units from the actual recording showed an increased neural response, however, most units from the LN prediction showed a decreased response. Although the neural response from the LN prediction showed changes over the stimulus transitions, often the change of LN predictions was opposite from that of the actual neural response or the change scale was different than for the actual neural response. This suggests the neural sensitivity to the statistical features is a nonlinear transformation instead of a simple linear dependence on the sound spectrogram change.

**Figure 8:**
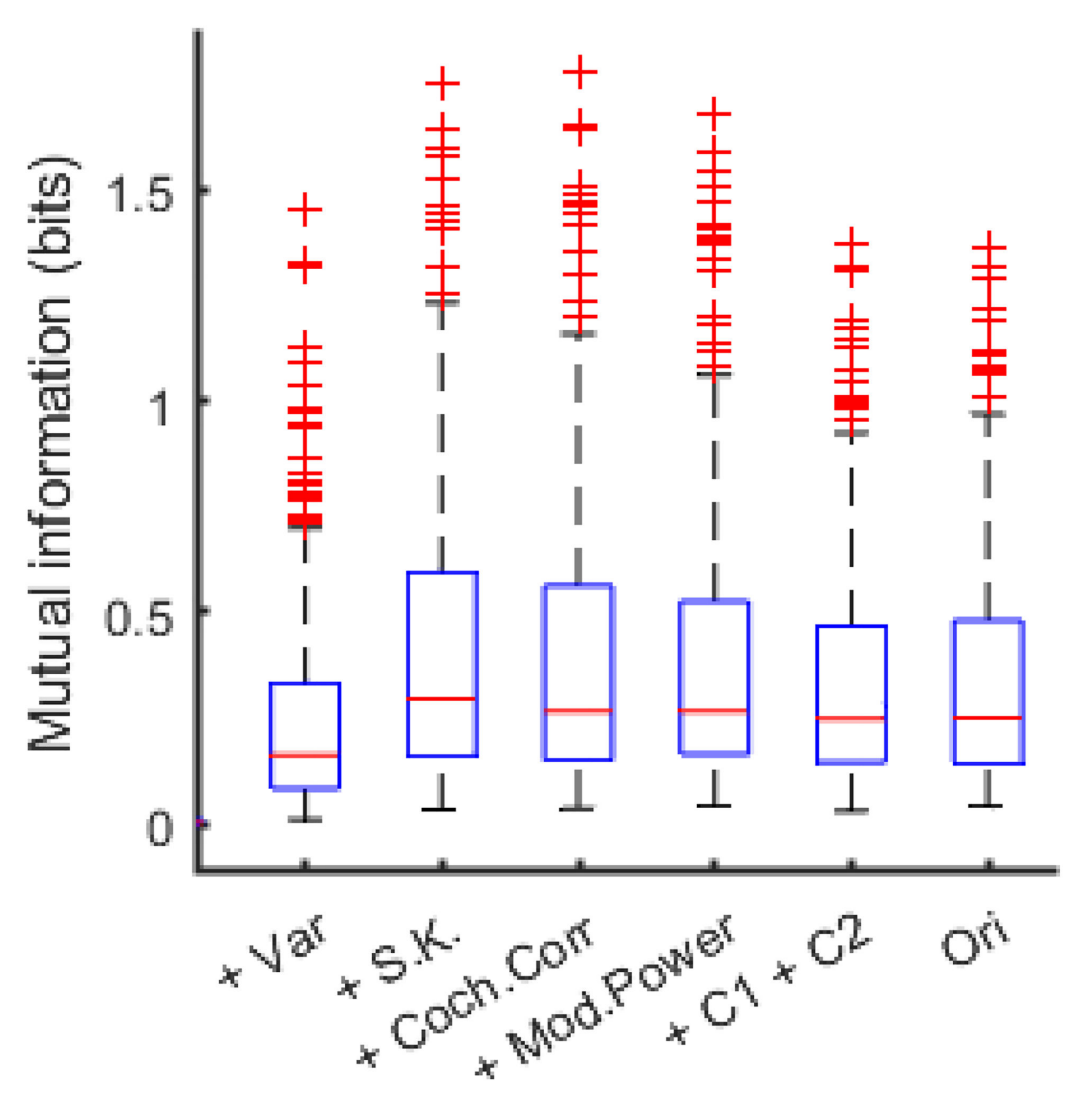
The MI distribution over multiunits for each synthetic variant. The red bar indicates the median MI over units (n = 387), and the edges indicate the 25th and 75th percentiles, respectively. The whiskers indicate the outliers.

The neural coding of the texture type was quantified by the MI of the sustained response for each synthetic variant. We found that the median of the MI values over units was 0.16 bits/response at the Power transition, 0.30 bits/response at +Var transition, 0.27 bits/response at +S.K, 0.27 bits/response at +Coch.Corr, 0.25 bits/response at +Mod.Power, 0.25 bits/response at +C1+C2, and 0.32 bits/response at +Ori, respectively. One might expect the MI values to increase progressively as more potentially identifying statistical features are added. However, we saw only a clear increase at the Power to +Var transition and then MI values appeared to plateau. That observation is perhaps not too surprising. The MI method here only quantifies sustained, steady-state responses over 1 s durations, which is unlikely to be well matched to the coding strategy employed by IC neurons, and as seen in Figure 4, it can also happen that between-exemplar variance increases with increasing complexity of texture features. As such, the plateau in the median MI values observed in Figure 8 is unsurprising if we assume that most IC neurons are not set up to encode texture type in steady state response amplitudes.

## 4 Discussion

In this study, a set of morphed stimuli was used to examine how commonly responses of IC neurons are sensitive to the statistical parameters known to characterize different types of environmental sound textures. This study has focused specifically on what proportions of the multiunits respond to the different statistical parameters present in the stimuli. How sensitivity to these statistical parameters arises in the brain is beyond the scope of the research question raised here but opens more interesting scientific questions for future studies.

We found that most multiunits in the IC are sensitive to most of the types of statistical features extracted by the auditory model from the study of McDermott et al. (2011). While some of these results are perhaps unsurprising, given for example that neurons in the IC are known to be sensitive to modulation (Frisina, 2001), other aspects are perhaps less expected. For example, it is not obvious why so many of the often narrowly frequency-tuned IC neurons should be sensitive to cochlear correlations. Nevertheless, we found that almost half of IC neurons will respond with an onset transient response to sudden changes in cochlear correlations, and almost 80% may respond to changes in correlations with changes in sustained firing rates (compare Figure 5).

It has long been thought that a statistically efficient representation of environmental information may be a design principle that guided the evolution of sensory systems (Attneave, 1954). The statistical structure of natural signals is highly conserved across natural sounds (Attias & Schreiner, 1997; Escabi, Miller, Read, & Schreiner, 2003; Nelken, Rotman, & Yosef, 1999; Singh & Theunissen, 2003; Voss, 1975). Both peripheral and central auditory neurons appear to match their response properties to statistical regularities in the acoustic environment to efficiently encode natural sounds (Attias & Schreiner, 1998; Escabi et al., 2003; J. A. Garcia-Lazaro, Ahmed, & Schnupp, 2006; Jose A Garcia-Lazaro, Ahmed, & Schnupp, 2011; Holmstrom, Eeuwes, Roberts, & Portfors, 2010; Lesica & Grothe, 2008; Nelken et al., 1999; S. M. N. Woolley, 2005) or to efficiently predict the immediate future of natural sounds (Singer et al., 2018). In the auditory pathway, the IC is an obligatory station that receives convergent inputs from numerous brainstem structures and sends its highly processed outputs to the auditory thalamus, and, subsequently, to primary auditory cortex. Numerous studies have reported that IC neurons are sensitive to various spectral and temporal stimulus attributes (Escabí & Schreiner, 2002; Irvine & Gago, 1990; Krishna & Semple, 2000; Kuwada et al., 1997; Langner & Schreiner, 1988; Ramachandran, Davis, & May, 1999; Rees & Møller, 1983, 1987; C. E. Schreiner & Langner, 1988; C. Schreiner, Urbas, & Mehrgardt, 1938). However, many such studies use only highly simplified stimuli, such as amplitude modulated tones or noise stimuli, which simplifies stimulus design and data interpretation, but the ecological validity of such simplified stimuli may be limited, raising questions about whether or how findings generalize to the much more complex natural soundscapes experienced by animals in the real world. The great diversity and complexity of natural sounds creates a barrier to analyzing neural responses to these sounds (Attias & Schreiner, 1998). Natural or naturalistic sound textures of the type used here may help us overcome some of these limitations since they contain much of the richness and diversity of ecological sounds recorded in nature, but they do remain fully described by a limited, albeit large, set of parameters (McDermott & Simoncelli, 2011).

Using the methodology developed by McDermott & Simoncelli (2011), we previously computed and analyzed the marginals, cochlear correlations, modulation power and modulation correlation statistics of 200 natural sound textures and subjected them to principal component analysis in order to reduce the complexity of McDermott & Simoncelli’s original high-dimensional feature space (Mishra et al., 2020). The sound textures used in this study were selected so as to cover a wide range of the resulting PC space of our corpus of natural sound recordings (Figure 1). We then used the generative model by McDermott et al. (2011) to create a set of synthetic stimuli which morph in a series of discrete transitions from flat, spectrally shaped noise to full exemplars of natural sound texture stimuli. By looking for changes in neural firing induced by each of these stepwise transitions we were able to determine whether mulitunits recorded in the IC were sensitive to the corresponding set of statistical features.

We found that at least some IC multiunits are sensitive to all the statistical features of natural sound textures described by McDermott & Simoncelli (2011), that most neurons were sensitive to most statistical features, and that such sensitivity could manifest through transient responses, or, more commonly, through changes in sustained mean firing rates. These differential transient vs sustained responses by the IC multiunits is reminiscent of a previous study by Zheng & Escabi (2008), which has also reported a differential encoding of sound envelope properties through transient and sustained responses. As a particular case in point, consider responses to the +S.K. transition. When +S.K. stimuli are synthesized from white noise, then these are very low on cochlear correlations and mostly sound somewhat like bubbling water (McDermott & Simoncelli, 2011). Only about ~6% IC multiunits signal a +S.K. transition with a transient response, but over half of them will respond with significant changes (usually declines, see Figure 6) in their sustained firing rates in response to at least some of the textures tested.

For the +Coch.corr statistical transition, 77% of the IC multiunits showed significant response in a sustained window whereas ~38% were sensitive in the transient window. The IC, due to its central location in the auditory pathway, receives convergent inputs from multiple brainstem structures. The IC neurons have also been reported to perform temporal integration (Voytenko & Galazyuk, 2007). Certain “higher order” statistics may require more time to be detected by central auditory neurons. A case in point are modulations. Ecologically relevant amplitude or frequency modulations can occur at relatively low modulation frequencies, and analysis windows cannot be shorter than the periods of these modulation frequencies if the strength of modulation to such low frequencies is to be determined. Numerous previous studies have documented the selectivity of IC neurons to spectrotemporal modulations (Escabi et al., 2003; Theunissen, Sen, & Doupe, 2000; Sarah M N Woolley et al., 2005) and have highlighted the importance that such modulations can have as information-bearing attributes (Elliott & Theunissen, 2009; Singh & Theunissen, 2003). Given this well documented importance of modulations, it is unsurprising that a high proportion of IC multiunits in our study were sensitive to the +Mod.Power transition, but it is interesting that as many as ~60% of the IC multiunits signaled that transition already in their transient response window, within 50 ms from the transition. Perhaps this widespread and surprisingly rapid sensitivity to changes in modulation parameters relates to observations by Zheng & Escabi (2008), who have described that IC neurons can be sensitive to the shape, and not just the rate, of modulation envelopes, and abrupt shape changes might be detectable quite rapidly. However, the extent to which such mechanisms apply to usually quite stochastic texture stimuli where exact repetitions of envelope shapes would not normally be expected will need to be investigated in the future.

We found most of the IC multiunits (~85%) to be sensitive to the +Ori in sustained windows. Although McDermott & Simoncelli’s (2011) psychophysical results in humans demonstrated that the synthesized textures with all subsets of texture parameters were often easily identifiable and highly realistic, our results nevertheless found that the large majority of IC multiunits were sensitive to the transition from the fully morphed texture with all parameters to a segment of the natural sound texture recording. A possible explanation for this is that there are additional features beyond those identified by McDermott & Simoncelli’s (2011) which distinguish real textures from synthetic ones, and which IC neurons are commonly sensitive to, but other possible explanations cannot be discounted. For example, Bar-Yosef & Nelken (2007) described that auditory cortex neurons can be exquisitely sensitive to rather arcane features of natural sound recordings, such as reverberant echoes, which listeners are often completely unaware of, and which a resynthesis of sound textures from statistical parameters with McDermott & Simoncelli’s toolbox would not reproduce. Responses of IC neurons are known to be strongly influenced by features such as reverberation (Slama & Delgutte, 2015). Previous neuroimaging studies in humans and ferrets showed that primary areas of the auditory cortex (A1) are not substantially sensitive to higher-order statistics distinguishing sounds synthesized from the acoustic model and natural sounds (Landemard et al., 2020; Norman-Haignere & McDermott, 2018). That might suggest that there are less neurons in A1 which are sensitive to the higher-order features not captured by the acoustic model. However, neuroimaging studies measure the population neural response at a slow time resolution (~2 s). If there are many A1 neurons which respond transiently, this gets smeared out in imaging with non-responsive segments. Therefore, imaging might not perfectly predict electrophysiological activity. Although the complex computation ability of IC was reportedly less than that of A1, the accuracy of coding the input spectrogram in IC is stronger than in A1 (Rabinowitz, Willmore, King, & Schnupp, 2013) and certain details of cochlea or brainstem stimulus representations may not to be transmitted to A1(Rahman, Willmore, King, & Harper, 2020). Thus, IC responses may be more strongly affected by sound spectrogram changes than are A1 neural responses, and we might expect IC neurons to exhibit a more obvious sensitivity to the higher-order stimulus statistics. Furthermore, the textures used in this study differ from those used in the neuroimaging studies (Landemard et al., 2020; Norman-Haignere & McDermott, 2018) and some textures may be less well captured by McDermott & Simoncelli’s model. Additionally, their model is optimized for human perception, and the rat might be sensitive to the other statistics beyond the statistical features in the acoustic model.

We found that most IC units were sensitive to the transitions of statistical features, however, the stimulus cochleagrams also changed as statistical features were added. We asked how much of this sensitivity could be explained by the neurons’ linear spectrotemporal tuning being applied of the changing cochleagrams. To this end, we built an LN model that we fit to the neural responses; this model consisted of a linear spectrotemporal receptive field and a simple static nonlinearity. The neural response predicted by the LN model showed changes over the stimulus transitions, however, often the changes of the LN predictions were opposite from those of the actual neural response or the change scale was different than for the actual neural response. This suggests that the neural sensitivity to the statistical features is a relatively complex nonlinear transformation, instead of a simple linear dependence on the sound spectrogram change. Our findings here with textures are consistent with previous findings in other contexts that also suggest that IC neural responses cannot be fully accounted for by a linear integration model (Escabı & Schreiner, 2002; Lee, Schrode, & Bee, 2017; Lyzwa, Chen, Escabi, & Read, 2020). Hence, our results suggest that there are nonlinear mechanisms impacting IC responses that enable sensitivity to the statistical features of textures.

We also undertook some investigation of the capacity of the IC neural responses to discriminate between the different texture types. Previous work on discrimination of five different texture stimuli suggests that IC has this capacity (Sadeghi et al., 2019; Zhai et al., 2020). Here for our 13 textures, we estimated MI to examine the neural coding of the texture type for each synthetic variant with varying statistical features and original sounds. From the human behavior results in McDermott & Simoncelli’s (2011) study, one might expect the MI values increase as more statistical features were added in synthetic variants. However, our results showed that the MI value increased from Power to +Var, and then reached a plateau. Several possible reasons might explain the seeming inconsistency between our neural data with the human behavior results (McDermott & Simoncelli, 2011). We quantified MI using the averaged sustained response over 1 s duration at a single trial level, so the MI value was affected by the trial-to-trial variability. The stimulus spectrogram becomes more dynamic after the + Coch. Corr transition (Figure 2 and Figure 3), which might increase trial-to-trial variability. For the human behavior task in McDermott & Simoncelli’s (2011) study, the listeners were asked to pick one texture name from five names after listening to one sound. The task was to match the sound to the participants’ known sounds. The MI values measured the discriminability across the 13 textures in the current study, which is different from the human behavior task.

A final interesting, but open, question is how our results relate to the large and growing literature on change detection in the auditory system. Deviance detection is thought to be adopted by the auditory system to give perceptual saliency to acoustic events that were not predictable based on sustained stimuli, and which therefore signal changes in the environment which may require a behavioral response (Winkler & Schröger, 2015). Two types of physiological signatures are often used to study neural correlates of auditory change detection: stimulus specific adaptation (SSA), which is usually quantified as the index of change in the firing rate of a neuron in response to a deviant stimulus when compared with its response to that same stimulus played as a standard (Nelken & Ulanovsky, 2007; von der Behrens, Bauerle, Kossl, & Gaese, 2009), and mismatch negativity (MMN), which is detectable in non-invasive scalp recordings. MMN has a much slower time course than SSA and is thought to be of cortical origin, but SSA at lower levels of the auditory pathway may facilitate MMN, and SSA has been observed as early as non-lemniscal parts of the IC (Aguilar Ayala & Malmierca, 2013; Anderson & Malmierca, 2013; Liberman, Epstein, Cleveland, Wang, & Maison, 2016; Malmierca, Cristaudo, Pérez-González, & Covey, 2009). SSA and MMN are often studied with highly regular, rhythmic stimulation, which makes the stimuli easy to parameterize but limits ecological validity. However, Winkler et al. (2003) observed MMN even in highly naturalistic stimuli that lacked regular rhythms, and it seems possible that the auditory system may achieve change detection by monitoring features of the acoustic environment which go beyond detecting a change in regular rhythmic patterns. Auditory textures are fundamentally noise-like and characterized by statistical distributions of features rather than highly regular patterns. It is interesting to speculate that transient responses to changes in statistical features of sustained sounds which we observed so frequently in IC responses, might represent another important change detection mechanism in the ascending auditory pathway, but this idea will require proper development in follow-on studies.
